# Ultrafast Laser Direct Writing of Diffraction Gratings in PVA/AA Photopolymer with Tunable Phase Modulation Mechanisms

**DOI:** 10.3390/polym18010046

**Published:** 2025-12-24

**Authors:** Andrés P. Bernabeu, Juan Carlos Bravo, Joan Josep Sirvent-Verdú, Belén Nieto-Rodríguez, Daniel Puerto, Sergi Gallego

**Affiliations:** 1 Departmento de Física, Ingeniería de Sistemas y Teoría de la Señal, Universidad de Alicante, 03690 San Vicente del Raspeig, Spain; juanc.bravo@ua.es (J.C.B.); jj.sirvent@ua.es (J.J.S.-V.); b.nieto@ua.es (B.N.-R.); dan.puerto@ua.es (D.P.); sergi.gallego@ua.es (S.G.); 2 I.U. Física Aplicada a las Ciencias y las Tecnologías, Universidad de Alicante, 03690 San Vicente del Raspeig, Spain

**Keywords:** femtosecond direct laser writing, diffraction gratings, PVA/AA photopolymers, high repetition rate, surface structuring, refractive index modulation

## Abstract

Diffractive optical elements (DOEs) are essential components in optical and photonic technologies, motivating the need for rapid mass production with high-precision fabrication methods. Photopolymers are particularly attractive for DOEs production because their optical phase can be modified via both surface and refractive index modulations. In this work, we report the fabrication of DOEs on PVA/AA photopolymers using ultrafast laser direct writing. By combining surface topography measurements with a phase depth model, we extract the surface and bulk phase depth contributions and demonstrate a transition from surface-dominated modulation at 1 kHz to bulk refractive index modification under cumulative conditions at 100 kHz. These outcomes highlight ultrafast laser direct writing as a powerful, rapid and controlled method for the high-quality and rapid fabrication of DOEs.

## 1. Introduction

Photopolymers are versatile materials mainly used in holographic applications. The initial chemical formulations were liquid at room temperature [1]. A second step in their development was the proposal of new formulations that remained solid at room temperature [2]. In this case, the basic composition of photopolymer consists of a binder that provides consistency and mechanical stability, within which the monomer (mono or poly functional) is dissolved together with a dye that absorbs the light in the desired wavelength and an electron donor compound that transfers the energy from the excited dye to generate free radicals in the illuminated regions [3,4]. Based on this fundamental composition, new additives have been incorporated to improve the material properties for specific applications. For example, crosslinkers have been used to increase the refractive index modulations in the illuminated zones [5], nanoparticles to reduce the shrinkage [6], and liquid crystal or magnetic molecules to generate dynamic holograms [7,8]. Some of these compositions have become commercially available for holographic purposes [9,10].

One well-established family of photopolymers is based on polyvinyl alcohol/acrylamide (PVA/AA). Initially, these compositions were also liquid [11], but later solid formulations were developed to obtain more stable layers [12,13]. Throughout their evolution, it was demonstrated that PVA/AA photopolymers exhibit very good behavior for recording low spatial frequency diffractive optical elements (DOEs) using a continuous wave laser at 532 nm [14], due to both refractive index modulation [15] and surface modulation [16]. Several research groups have contributed additional characterizations for recording this type of low spatial frequency elements in such materials [17,18,19], and similar effects have been demonstrated in other photopolymers that also exhibit good behavior in this spatial frequency range [20,21,22].

Different techniques have been used to generate modulated DOEs onto photosensitive materials. Since photopolymers respond to light intensity, an amplitude DOE projected onto the material is transformed into a phase DOE during recording. Illumination methods include small-angle interference between two coherent beams, micromirror arrays [20], or Liquid Crystal on Silicon (LCoS) devices [23]. Pixelated modulators offer the advantage of enabling dynamic DOEs without requiring modifications in the experimental setup, although the pixelation must be removed in the optical recording system with a low-pass filter, introducing a reduction in the effective resolution. Despite this limitation, complex DOEs have been successfully fabricated using these approaches [23].

The importance of the DOEs has increased in recent years due to applications in ophthalmology, intraocular lenses, augmented reality, novel camera systems, and visible and infrared sensors technology [24,25,26]. In this context, photopolymers are attractive materials for fabricating phase DOEs, as both surface relief and refractive index modulation mechanisms can contribute to phase control. Surface relief enables higher phase contrast, as surface variations in the micron scale are achievable, while refractive index modulation is around 10^−3^. Considering that layer thicknesses range between 40 and 800 μm, surface modulation provides a larger phase shift, although refractive index modulation tends to be more stable.

Polymers offer many advantages as optical materials, including low cost and the possibility of fabricating a large number of elements through mass production. Although their low absorption in the visible spectrum is also advantageous for optical applications, additional approaches such as the recording of DOEs using UV light or ultrafast laser processing have been explored [27,28,29]. Indeed, ultrafast laser processing of polymers is a relatively recent field, in which the generation of DOEs in different polymeric materials, such as PDSM [30], PMMA and PMMI [31], and the generation of ablative structures in PVC, PET and PP [32,33], have already been reported.

Photopolymers represent a special class of polymers due to their intrinsic light sensitivity. Previous works have analyzed the polymerization dynamics of photopolymers under pulsed laser exposure [34], and more specifically, the phase shift modulation achievable in PVA/AA using high repetition rate pulsed illumination [35]. In the context of holographic recording, early studies demonstrated the formation of diffraction gratings in PVA/AA using nanosecond pulsed lasers, reporting moderate to high diffraction efficiencies depending on the material composition and recording conditions [36,37]. More recently, highly efficient holographic gratings in PVA/AA have also been obtained using nanosecond coherent illumination [38]. Despite these advances, previous works have primarily focused on pulsed sources in the nanosecond regime, without investigating the separate contributions of surface relief and bulk refractive index changes to the resulting phase modulation. Furthermore, the use of ultrashort laser writing at high repetition rates for the fabrication of DOEs in PVA/AA has not been explored in detail.

In the present work, we propose the fabrication of DOEs using ultrafast laser direct writing, a technique that avoids the pixelation associated with spatial light modulators and provides high flexibility, as the induced phase shift can be controlled through the irradiation conditions. The high precision of the scanners used in the ultrafast laser direct writing systems enables high control over the writing geometry, making this approach suitable for fabricating high-precision DOEs.

To this end, we have recorded diffraction gratings over a wide range of fluence at two different repetition rates. The surface profiles of the induced structures were measured directly using an optical profilometer. In previous studies, surface profiles have been inferred from the diffraction efficiencies of the different diffracted orders to estimate the most probable surface relief [39]. In this work, we combine the measured diffraction efficiencies and the experimentally determined surface profiles to estimate the optical and the surface phase depth contributions of the gratings, and to analyze their dependence on fluence in the non-cumulative and cumulative irradiation regimes.

## 2. Materials and Methods

The laser processing system consists of an ultrashort source (NKT aeroPULSE FS50, Birkerød, Denmark) delivering 450 fs pulses at three wavelength configurations (λ = 1030, 515 and 343 nm). The maximum output power at 1030 nm is 50 W, and the repetition rate can be tuned from 500 Hz and 1 MHz. The beam positioning on the sample surface is controlled by a scanner system equipped with two movable mirrors, enabling the fabrication of arbitrary geometries over the X-Y plane. An F-theta lens with a 170 mm effective focal length and a 7 × 7 cm^2^ scanning field ensures uniform focusing across the entire processing area. The beam radius at the focal plane for 515 nm is 9 µm at 1/e^2^, determined with Liu’s method [40].

**Figure 1:**
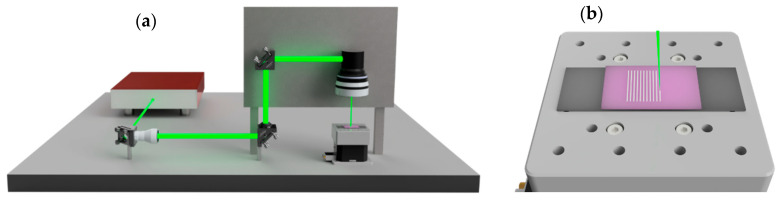
(**a**) Schematic representation of the femtosecond laser direct writing system, including the optical elements, the scanner system and the F-theta lens used for the writing of the gratings. (**b**) Illustration of the grating writing method, showing the line scanning procedure on the photopolymer surface.

The grating fabrication was performed by adjusting the scanner velocity to deliver 1 pulse per micron at the selected repetition rate. The lateral spacing between consecutive scan lines was adjusted to define the grating period. A schematic description of the laser system and a sketch of the grating writing procedure are shown in Figure 1a,b, respectively.

The recording material is a custom prepared polyvinyl alcohol/acrylamide (PVA/AA) photopolymer. PVA acts as the polymeric binder, acrylamide (AA) as the monomer, triethanolamine (TEA) as the photoiniciator, N,N’methylene-bis-acrylamide (MBA) as the crosslinker and yellowish eosin (YE) as the dye. The concentrations of the components in the photopolymer solution are summarized in Table 1.

After the preparation, 500 µL of the photopolymer solution was deposited onto 2 × 2 cm^2^ glass microscopic slide substrates. The films were left to dry for 24 h at ambient conditions to ensure full solidification, resulting in films with a thickness of 114 µm, approximately.

The dried photopolymer layers were subsequently processed using the femtosecond laser system by writing the diffraction gratings at the material surface. After laser exposure, the samples were bleached under ambient light for 48 h to remove the residual dye.

**Figure 2:**
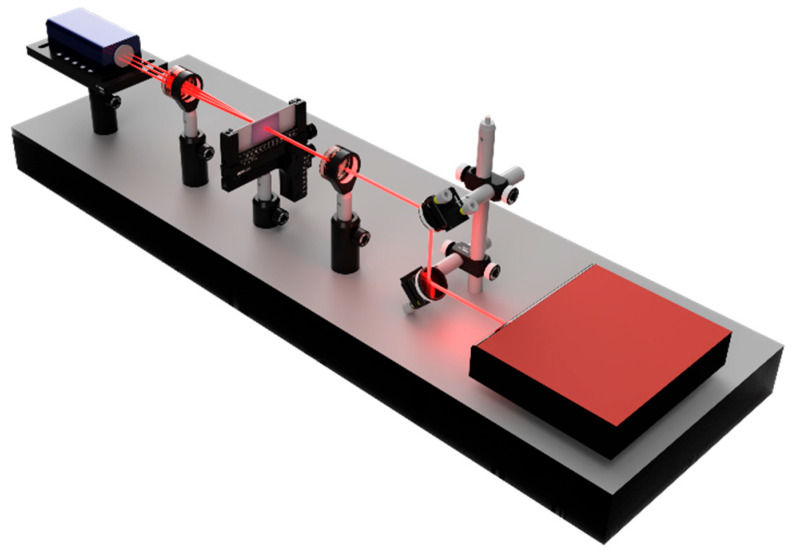
Schematic representation of the experimental setup used to measure the diffraction efficiencies of the fabricated gratings composed of a supercontinuum laser source, absorptive neutral density filters, the sample with the written grating, a 75 mm lens and a camera at the focal plane.

The diffraction efficiencies were measured using the setup presented in Figure 2. The system consists of a supercontinuum laser (NKT Photonics SuperK EVO, Birkerød, Denmark) coupled to a tunable wavelength selector (NKT Photonics SuperK VARIA, Birkerød, Denmark), adjusted to 633 nm. The laser beam passes through absorptive neutral density filters before reaching the fabricated gratings, generating a diffraction grating that is collected by a 75 mm focal length lens. A camera placed at the focal plane of the lens captures the intensity distribution of the diffraction orders. The diffraction efficiency was calculated as the ratio between the measured intensity of each order and the intensity of the incident beam.

The same measurements were repeated several days after the fabrication of the gratings, showing no significant deviation from the initial diffraction efficiencies.

The surface topography and the height modulation of the laser-induced structures were characterized using an optical profilometer (Filmetrics Profilm 3D, San Diego, CA, USA) equipped with a 20× interferometric objective (Nikon, NA 0.4, Tokyo, Japan), providing high-resolution surface measurements.

## 3. Results

As discussed above, femtosecond laser processing enables precise control of surface modifications, becoming a suitable tool for the fabrication of diffraction gratings on PVA/AA photopolymer layers. To investigate the influence of the laser parameters on the morphology and the optical response of these structures, different series of 3 × 3 mm^2^ gratings were fabricated using λ = 515 nm at two representative repetition rates, 1 kHz and 100 kHz.

These two repetition rates were intentionally selected to analyze two distinct thermal regimes. At 1 kHz, the time between pulses (1 ms) is large enough to achieve thermal relaxation between pulses, corresponding to a non-cumulative regime. Conversely, at 100 kHz, the shorter interval between pulses (10 μs) prevents the complete cooling of the material before the next pulse arrives, resulting in a cumulative regime where heat accumulation modifies the material response [32,33].

For each repetition rate, the fluence was varied between 0.006 and 0.89 J/cm^2^, with each grating written at a different combination of fluence and repetition rate. The gratings were produced writing line by line, adjusting the distance between lines equal to the Full Width at Half Maximum (FWHM) diameter of the single-line structure characterized beforehand [41]. Therefore, the effective grating period results from the lateral expansion of each written line, governed by the swelling response of the material. The number of pulses was kept constant to 1 pulse per micron (18 pulses per beam diameter) by adjusting the scanning velocity for each repetition rate. This ensured that accumulation was sufficiently achieved in spot diameter when processing at high repetition rates.

This transition between these thermal regimes is expected to be reflected in both the surface morphology and the optical behavior of the fabricated gratings.

### 3.1. Gratings Morphology

**Figure 3:**
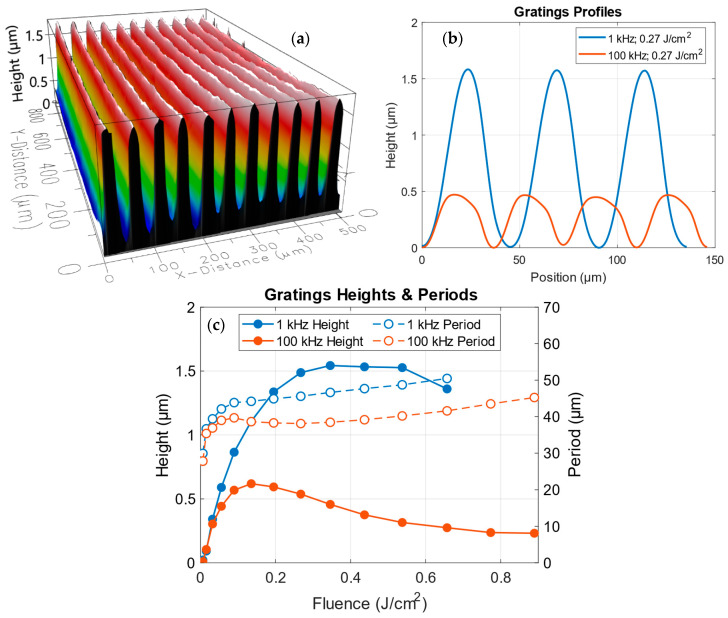
(**a**) 3D Interferometric profilometry image of a written grating at 1 kHz and 0.27 J/cm^2^. (**b**) Comparison of height profiles measured for gratings written at 1 kHz (blue) and 100 kHz (orange) at the same fluence (0.27 J/cm^2^). (**c**) Dependence of the maximum height (solid markers, left axis) and the grating period (open markers, right axis) on the laser fluence for 1 kHz (blue) and 100 kHz (orange).

The morphology of the fabricated gratings strongly depends on the laser fluence and repetition rate. Figure 3 shows the representative profile characteristics of the gratings obtained at 1 and 100 kHz for different fluences.

Figure 3a presents a 3D profilometric image of a grating (R = 1 kHz, F = 0.27 J/cm^2^), exhibiting a well-defined sinusoidal surface modulation. Figure 3b compares the height profiles for 1 kHz and 100 kHz written at 0.27 J/cm^2^.

The evolution of the periods of the gratings, also shown in Figure 3c (open markers, dashed lines), exhibits a similar saturating behavior.

At 1 kHz (Figure 3b, blue), the gratings exhibit a pronounced sinusoidal profile with clear periodic modulation and amplitudes larger than 1.5 μm. At 100 kHz (Figure 3b, orange), the surface modulation is notably smaller (<0.5 μm) and the shape deviates from the strict sinusoidal form, exhibiting flatter tops, and even a slight asymmetry.

The dependence of the maximum height $h_{max}$ on the fluence for different repetition rates is shown in Figure 3c. The data show an initial increase in the $h_{max}$ with fluence, up to a saturation point followed by a slight decrease for both 1 kHz (blue solid makers, solid lines) and 100 kHz (orange solid markers, solid lines). A clear reduction of $h_{max}$ with repetition rate is observed, as notable smaller heights are achieved at 100 kHz. Moreover, the decrease in heights produced at greater fluences is more pronounced for the 100 kHz case. This suggests that cumulative heating suppresses the surface relief even when the same energy is delivered in each pulse.

The evolution of the spatial periods of the gratings is also shown in Figure 3c (open markers, dashed lines). As described above, the line spacing was set equal to the previously characterized FWHM of the single-line irradiations, exhibiting also a fluence-dependent saturating behavior.

An additional observation concerning the onset of the surface damage at low repetition rates must be discussed. While processing at 100 kHz allows the use of considerably higher fluences without degrading the surface morphology, the gratings written at 1 kHz above ~0.66 J/cm^2^ exhibit clear signs of surface degradation. A plausible explanation is that, in the non-cumulative regime, the energy deposited by each pulse remains confined to the regions near the surface region, leading to superficial material decomposition. In contrast, at 100 kHz, the cumulative heating redistributes the deposited energy over a larger and deeper volume, favoring bulk modification rather than localized surface damage.

**Figure 4:**
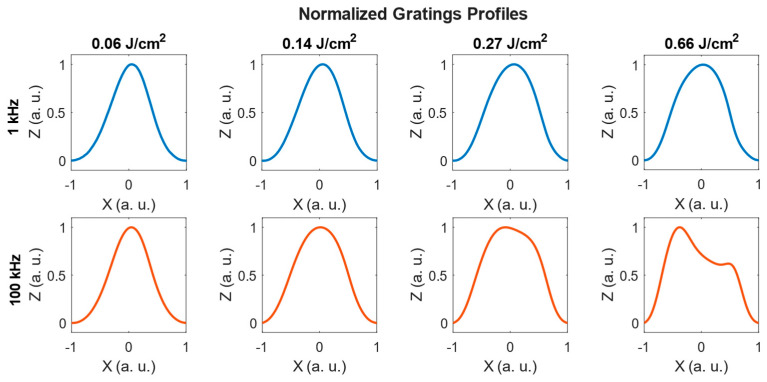
Normalized height profiles (Z) extracted from interferometric profilometry measurements for gratings written at 1 kHz (**top row**, blue) and 100 kHz (**bottom row**, orange), at four representative fluences (0.06, 0.14, 0.27 and 0.66 J/cm^2^).

Figure 4 compares the normalized profiles for several fluences at 1 kHz and 100 kHz. The heights have been normalized in arbitrary units between 0 and 1 for a better comparison of the shapes.

At 1 kHz, the profiles remain nearly sinusoidal across the whole fluence range, only slightly broadening at larger fluences (0.66 J/cm^2^). At 100 kHz, the profiles evolve from sinusoidal at low fluences (0.06 J/cm^2^) to broader and flatter shapes at moderate fluences (0.14–0.27 J/cm^2^), and finally to asymmetric forms at higher fluences (0.66 J/cm^2^) with secondary maxima beside the main peak. This effect indicates the onset of the splitting effect, previously described in [41], which may contribute to the reduction in the spatial period of the gratings.

These results demonstrate that the laser repetition rate is a powerful control parameter for tuning the grating morphology, from pronounced sinusoidal reliefs in the non-cumulative regime (1 kHz) to nearly flat or asymmetric shallower structures in the cumulative regime (100 kHz). At 1 kHz, the larger relief amplitudes suggest that surface modulation plays an important role in the diffractive response of the gratings. At 100 kHz, the reduced relief height implies that bulk refractive-index changes may be more determinant in the total phase modulation, as will be discussed in Section 3.3.

### 3.2. Diffraction Efficiencies

The diffraction efficiencies of the transmitted orders were measured under normal incidence using a He-Ne laser (λ = 633 nm).

**Figure 5:**
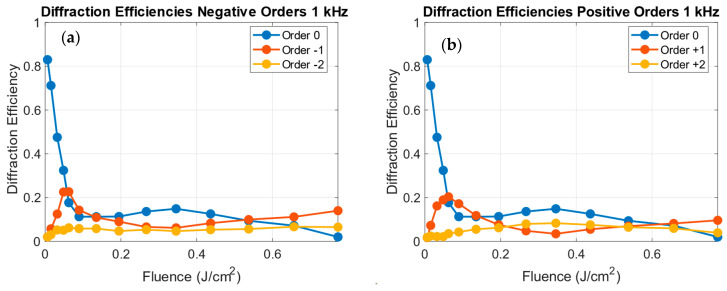
Diffraction efficiencies for the gratings written at 1 kHz for the negative orders (**a**) 0 (blue), −1 (orange) and −2 (yellow) and for the positive orders (**b**) 0 (blue), +1 (orange) and +2 (yellow) as a function of the fluence.

Figure 5 shows the diffraction efficiencies of the 0, ±1 and ±2 orders as a function of the fluence for the gratings written at 1 kHz. The intensities were normalized to the incident beam energy.

**Figure 6:**
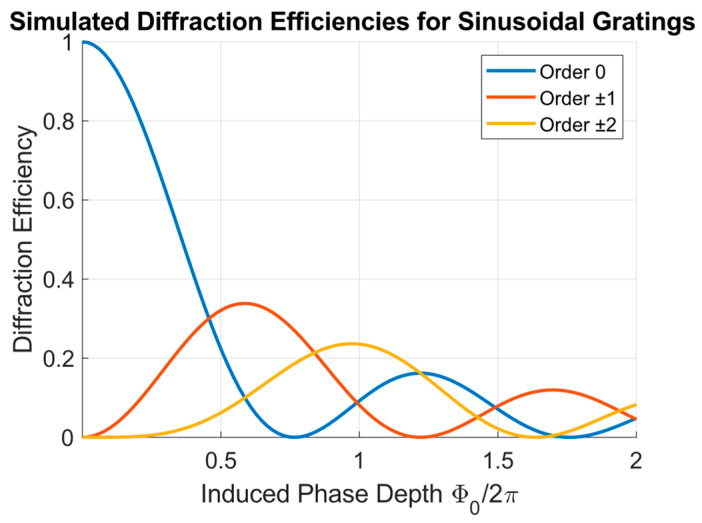
Calculated diffraction efficiencies for the 0, ±1 and ±2 transmitted orders for sinusoidal gratings as a function of the induced phase depth ($\phi_{0}$/2*π*).

As observed in Figure 5, at 1 kHz the ±1 and ±2 orders exhibit the expected symmetric behavior. Initially, as fluence increases, the diffraction efficiency of the ±1 orders rise to a maximum and then gradually decreases and stabilizes for fluences greater than 0.15 J/cm^2^. The ±2 orders also increase for low fluence values, and then remain weaker and nearly constant throughout the range. This trend is consistent with the theoretical dependence of the diffraction efficiency on the phase depth $\phi_{0}$ for sinusoidal phase gratings, as depicted in Figure 6.

Figure 6 presents the theoretical diffraction efficiencies for an ideal sinusoidal phase grating as a function of $\phi_{0}$, calculated for the 0, ±1 and ±2 orders. The curves were obtained from the Fourier decomposition of a sinusoidal phase profile.

The comparison with the experimental data at 1 kHz reveals a good qualitative agreement. The clear appearance of the experimental ±1 diffraction peak and the increase in the ±2 orders for fluence values up to 0.2 J/cm^2^ indicate that the 1 kHz gratings behave as nearly sinusoidal phase gratings, where the first-order efficiency peaks with a value of 0.33 for $\phi_{0}$ ≈ 0.57 (in 2π units). For fluences above 0.2 J/cm^2^, the diffraction efficiencies stabilize, suggesting that the induced phase depth saturates as the surface relief amplitude reaches its maximum, consistent with the morphological data of Figure 3.

**Figure 7:**
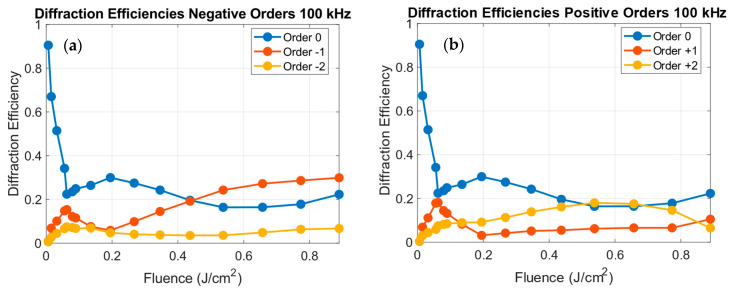
Diffraction efficiencies for the gratings written at 100 kHz for the negative orders (**a**) 0 (blue), −1 (orange) and −2 (yellow) and for the positive orders (**b**) 0 (blue), +1 (orange) and +2 (yellow) as a function of the fluence.

At 100 kHz the diffraction pattern differs notably, as presented in Figure 7.

As observed in Figure 7, at 100 kHz the ±1 orders are slightly weaker for fluences below 0.2 J/cm^2^, and a pronounced asymmetry between the positive and the negative orders appears at higher fluences. The enhancement of the −1 and +2 orders suggests that the asymmetrical surface shape redistributes the energy among the orders, approaching the theoretical maxima for sinusoidal gratings. These variations in diffraction efficiency directly correlate with the morphological evolution discussed in Section 3.1, as smaller and asymmetrical profiles are achieved at higher repetition rates and fluences. This suggests a potential regime transition from pure surface relief to mixed surface and optical modulation gratings under cumulative irradiation conditions.

### 3.3. Phase Depth Model and Fitting Analysis

The combined analysis of the diffraction efficiencies and the surface profiles provides deeper insights into the type of modifications that are induced on the material under different thermal regimes. While the morphological measurements reveal the external shape of the gratings, the optical response depends both on the surface relief and the internal refractive index variations.

To quantify the relative contributions of surface relief and bulk refractive index modulation, we applied a phase depth model linking the measured phase profiles and the diffraction efficiencies.

The induced phase modulation is expressed as:

(1) $$\phi \left(x\right)=\;\frac{2\pi }{\lambda }\left[d\Delta n_{pol}+h_{max}\left(n_{mat}-1\right)\right]H\left(x\right)=\phi_{0}H\left(x\right),$$

where λ is the reconstruction wavelength (633 nm), d is the material thickness (100 µm), $\Delta n_{pol}$ represents the index change between the illuminated and non-illuminated area due to bulk polymerization, $h_{max}$ is the maximum height of the measured profile of the grating and $n_{mat}$ is the refractive index of the unmodified material (1.4679 [15,42]). We assume that the phase modulation follows the normalized periodicity of the measured profile of the grating H(x) (that takes values between −1 and +1), and $\phi_{0}$ is the phase depth amplitude.

This equation allows us to identify two different contributions to the phase depth:

(2) $${\phi_{0}}_{surf}=\;\frac{2\pi }{\lambda }h_{max}\left(n_{mat}-1\right)$$

(3) $${\phi_{0}}_{opt}=\frac{2\pi }{\lambda }d\Delta n_{pol}$$

The surface term (${\phi_{0}}_{surf}$) is directly computed from the measured topography. The optical term (${\phi_{0}}_{opt}$) is obtained from the fitting of the single free parameter $\phi_{0}$ in Equation (1), using the measured H(x) as fixed input.

In practice, we simulate the diffraction efficiencies of the orders m ($\eta_{m}={\left|T_{m}\right|}^{2}$) by Fourier decomposition of the transmission function $t\left(x\right)=\;e^{i\phi (x)}$, selecting the $\phi_{0}$ values that minimize the deviation between the measured and the simulated diffraction efficiencies for the 0, ±1 and ±2 orders.

The Fourier coefficients are given by:

(4) $$T_{m}=\frac{1}{L}\int_{0}^{L}t(x)e^{-i\frac{2\pi mx}{L}}dx,$$

where $\phi_{0}$ was the only adjustable parameter, ensuring physical continuity with fluence and avoiding branch jumps in the periodic theoretical functions. Once $\phi_{0}$ was obtained, the optical phase contribution was deduced as ${\phi_{0}}_{opt}=\phi_{0}-{\phi_{0}}_{surf}$.

**Figure 8:**
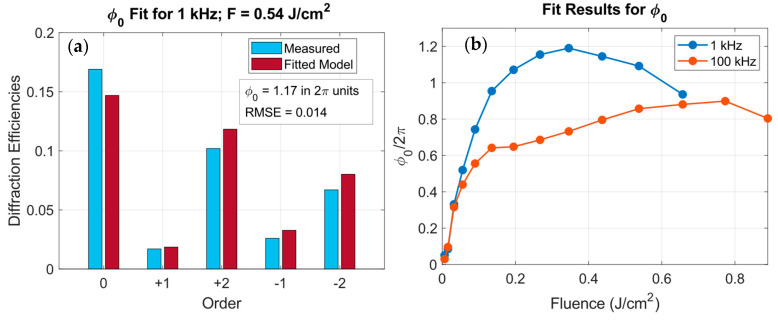
(**a**) Example of the fitted (red) and experimental (blue) diffraction efficiencies for a grating fabricated at 1 kHz and 0.54 J/cm^2^. (**b**) Evolution of the fitted phase depth $\phi_{0}$/2*π* as a function of the fluence for 1 kHz (blue) and 100 kHz (orange).

Figure 8a shows an example of the fitted and experimental efficiencies for a 1 kHz grating at 0.54 J/cm^2^, with a significant agreement between both curves as quantified by the root mean square error (RMSE = 0.014). The evolution of the fitted $\phi_{0}$ with fluence for both repetition rates is plotted in Figure 8b.

As depicted in Figure 8b, at 1 kHz, $\phi_{0}$ increases with fluence up to ~0.2 J/cm^2^, stabilizing around $\phi_{0}$ ≈ 1 (2π units) and slightly decreasing at higher fluences. The measured peak of $\eta_{1}$ (see Figure 3) occurs at F ≈ 0.05 J/cm^2^, corresponding to $\phi_{0}$ ≈ 0.5 (2π units), matching with the theoretical maximum of a sinusoidal grating depicted in Figure 6. At 100 kHz, two regimes are distinguished: below 0.2 J/cm^2^, $\phi_{0}$ follows a similar trend but with smaller values. Above this threshold, the slope reduces, coinciding with the onset of the flatter and asymmetric morphologies and the enhanced −1 and 2 orders. Therefore, from Figure 8b, we can infer that for repetition rates in the non-cumulative regime, higher phase depths can be achieved compared to higher repetition rates.

### 3.4. Surface and Optical Phase-Depth Contributions

**Figure 9:**
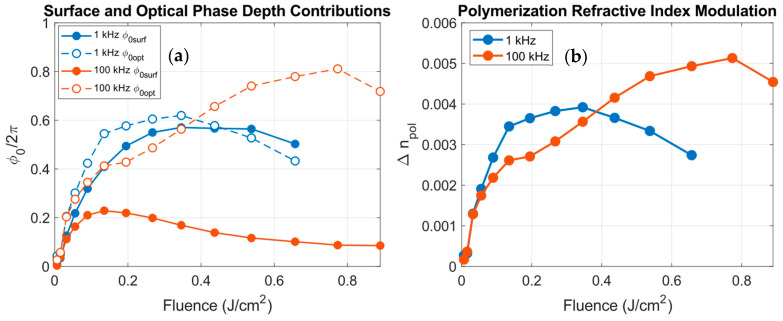
(**a**) Computed surface ($\phi_{0_{surf}}$, solid markers) and fitted optical ($\phi_{0_{opt}}$, open markers) phase depth components for gratings fabricated at 1 kHz (blue) and 100 kHz (orange) as a function of the fluence. (**b**) Refractive index modulation due to polymerization obtained from the fit as a function of the fluence at 1 kHz (blue) and 100 kHz (orange).

The main advantage of this analysis is that it allows us to quantify the relative contribution of both bulk optical modifications and surface relief modulations to the total phase depth, as well as their impact on the diffraction efficiencies of the written gratings. These contributions are presented in Figure 9.

At 1 kHz, the surface contribution (blue points, solid lines) and the optical modulation (blue circles, dashed lines) are comparable for all the fluence range, indicating that both relief and internal index modulation coexist in the non-cumulative regime. At 100 kHz, however, the surface contribution (orange points, solid lines) is significantly suppressed, while the optical contribution (orange circles, dashed lines) becomes dominant and increases with fluence. This behavior confirms that processing in the cumulative regime favors bulk refractive index modulations, while at low repetition rates, the material response remains largely confined to the surface.

It is also instructive to analyze the refractive index modulation due to photopolymerization ($\Delta n_{pol}$) associated with the bulk contribution ${\phi_{0}}_{opt}$. The values of $\Delta n_{pol}$ obtained from the fit as functions of the fluence for both 1 and 100 kHz are shown in Figure 9b. Since $\Delta n_{pol}$ is proportional to the bulk phase contribution, its evolution with fluence follows the same trends already discussed for Figure 9a. The maximum index modulations fall in the range of 5 × 10^−3^, in agreement with the expected polymerization refractive index modulations in PVA/AA photopolymers [5,38,43,44]. Moreover, these values are consistently higher at 100 kHz than at 1 kHz, confirming that cumulative heating enhances the bulk material modification and leads to a more efficient refractive index increase.

These results demonstrate that adjusting the repetition rate provides an effective way to tune the balance between the surface (${\phi_{0}}_{surf}$) and the bulk (${\phi_{0}}_{opt}$) phase depth contributions. At low repetition rates, the process is relief-dominated, whereas at high repetition rates, the process transitions to a bulk-dominated regime, where internal optical modulation governs diffraction.

This tunability opens a pathway for designing hybrid gratings with tailored optical responses through the appropriate selection of the irradiation conditions.

## 4. Conclusions

In this work, we have demonstrated an efficient method for the fabrication of diffractive optical elements (DOEs) on PVA/AA photopolymers using ultrafast laser direct writing. This technique enables flexible and rapid patterning over the material surface and allows control of the type of induced phase depth by simply adjusting the laser repetition rate. At low repetition rates, the process is relief-dominated, whereas at high repetition rates it transitions to a bulk refractive index modulation regime.

The refractive index modulations obtained in the cumulative regime reach values of $\Delta n_{pol}$ ~5 × 10^−3^, consistent with polymerization changes reported for PVA/AA photopolymers. This confirms that ultrafast irradiation can efficiently enable bulk photopolymerization, even when the beam is focused on the surface of the material, allowing the fabrication of high-quality DOEs.

Overall, these results establish ultrafast laser direct writing as a robust and versatile technique for manufacturing surface, bulk, or hybrid DOEs in PVA/AA photopolymers. Compared with DOEs fabrication using continuous wave lasers combined with spatial light modulators, the ultrafast laser writing technique provides high precision, offering complete freedom in the choice of the processing geometry while avoiding the pixelation artefacts. Beyond DOEsfabrication, the ability to induce relief modifications on the surface of the photopolymers opens new pathways for the functionalization of these materials, enabling arbitrary patterns of structures that may be exploited in biological and cell-guidance, microfluidic and wettability applications.

## Figures and Tables

**Table 1:** Concentrations of the components in the PVA/AA photopolymer formulation.

Compound	PVA	AA	MBA	TEA	YE
Concentration	8% *w*/*v*	0.501 M	0.055 M	0.351 M	0.196 mM

## Data Availability

The original contributions presented in this study are included in the article. Further inquiries can be directed to the corresponding author.

## References

[1] Close D.H., Jacobson A.D., Margerum J.D., Brault R.G., McClung F.J. (1969). Hologram recording on photopolymer materials. Appl. Phys. Lett..

[2] Sadlej N., Smolinska B. (1975). Stable photo-sensitive polymer layers for holography. Opt. Laser Technol..

[3] Guo J., Gleeson M.R., Sheridan J.T. (2012). A review of the optimisation of photopolymer materials for holographic data storage. Phys. Res. Int..

[4] Gallego S., Neipp C., Fernández R., Bravo J.C., Sirvent-Verdú J.J., Bernabeu A.P., Pascual I., Beléndez A. (2023). Modelling photopolymer behavior as optical recording medium. Asian J. Phys..

[5] Neipp C., Gallego S., Ortuño M., Márquez A., Beléndez A., Pascual I. (2003). Characterization of a PVA/acrylamide photopolymer: Influence of a cross-linking monomer in the final characteristics of the hologram. Opt. Commun..

[6] Tomita Y., Suzuki N., Chikama K. (2005). Holographic manipulation of nanoparticle distribution morphology in nanoparticle-dispersed photopolymers. Opt. Lett..

[7] Eich M., Wendorff J.H., Reck B., Ringsdorf H. (1987). Reversible digital and holographic optical storage in polymeric liquid crystals. Macromol. Rapid Commun..

[8] Irfan M., Martin S., Obeidi M.A., Miller S., Kuster F., Brabazon D., Naydenova I. (2022). A Magnetic Nanoparticle-Doped Photopolymer for Holographic recording. Polymers.

[9] Curtis K., Psaltis D. (1994). Characterization of the DuPont photopolymer for three-dimensional holographic storage. Appl. Opt..

[10] Bruder F.-K., Fäcke T., Rölle T. (2017). The Chemistry and Physics of Bayfol^®^ HX Film Holographic Photopolymer. Polymers.

[11] Calixto S. (1987). Dry polymer for holographic recording. Appl. Opt..

[12] Blaya S., Mallavia R., Carretero L., Fimia A., Madrigal R.F. (1998). Highly sensitive photopolymerizable dry film for use in real time holography. Appl. Phys. Lett..

[13] García C., Pascual I., Fimia A. (1999). Diffraction efficiency and signal-to-noise ratio of diffuse-object holograms in real time in polyvinyl alcohol photopolymers. Appl. Opt..

[14] Gallego S., Márquez A., Méndez D., Neipp C., Ortuño M., Beléndez A., Fernández E., Pascual I. (2008). Direct analysis of monomer diffusion times in polyvinyl/acrylamide materials. Appl. Phys. Lett..

[15] Gallego S., Fernández R., Márquez A., Ortuño M., Neipp C., Gleeson M.R., Sheridan J.T., Beléndez A. (2015). Two diffusion photopolymer for sharp diffractive optical elements recording. Opt. Lett..

[16] Gallego S., Márquez A., Ortuño M., Francés J., Marini S., Beléndez A., Pascual I. (2011). Surface relief model for photopolymers without cover plating. Opt. Express.

[17] Close C.E., Gleeson M.R., Sheridan J.T. (2011). Monomer diffusion rates in photopolymer material. Part I. Low spatial frequency holographic gratings. J. Opt. Soc. Am. B.

[18] Close C.E., Gleeson M.R., Mooney D.A., Sheridan J.T. (2011). Monomer diffusion rates in photopolymer material. Part II. High-frequency gratings and bulk diffusion. J. Opt. Soc. Am. B.

[19] Babeva T., Naydenova I., Martin S., Toal V. (2008). Method for characterization of diffusion properties of photopolymerisable systems. Opt. Express.

[20] Infusino M., Luca A.D., Barna V., Caputo R., Umeton C. (2012). Periodic and aperiodic liquid crystal-polymer composite structures realized via spatial light modulator direct holography. Opt. Express.

[21] Doskolovich L.L., Bezus E.A., Morozov A.A. (2018). Multifocal diffractive lens generating several fixed foci at different design wavelengths. Opt. Express.

[22] Osipov V., Pavelyev V., Kachalov D., Žukauskas A., Chichkov B. (2010). Realization of binary radial diffractive optical elements by two-photon polymerization technique. Opt. Express.

[23] Bravo J.C., Sirvent-Verdú J.J., Colomina-Martínez J., Reyna J., Fernández R., Márquez A., Gallego S. (2025). Multifocal Fresnel lenses with adjustable focal points recorded in photopolymers. Opt. Express.

[24] Shi K., Zhang G. (2022). Design and Application of Phase-Only Diffractive Optical Element Based on Non-Iterative Method. Photonics.

[25] Trung H.T.D., Ghim Y.-S., Rhee H.-G. (2025). Fabrication of Diffractive Optical Elements to Generate Square Focal Spots via Direct Laser Lithography and Machine Learning. Photonics.

[26] Pavelyev V., Khonina S., Degtyarev S., Tukmakov K., Reshetnikov A., Gerasimov V., Osintseva N., Knyazev B. (2023). Subwavelength Diffractive Optical Elements for Generation of Terahertz Coherent Beams with Pre-Given Polarization State. Sensors.

[27] Sola D., Alamri S., Lasagni A.F., Artal P. (2019). Fabrication and characterization of diffraction gratings in ophthalmic polymers by using UV direct laser interference patterning. Appl. Surf. Sci..

[28] Sola D., Cases R. (2020). High-Repetition-Rate Femtosecond Laser Processing of Acrylic Intra-Ocular Lenses. Polymers.

[29] Sola D., Aldana J.R.V.d., Artal P. (2020). The Role of Thermal Accumulation on the Fabrication of Diffraction Gratings in Ophthalmic PHEMA by Ultrashort Laser Direct Writing. Polymers.

[30] Park J.-K., Cho S.-H. (2011). Flexible gratings fabricated in polymeric plate using femtosecond laser irradiation. Opt. Lasers Eng..

[31] Wochnowski C., Cheng Y., Meteva K., Sugioka K., Midorikawa K., Metev S. (2005). Femtosecond-laser induced formation of grating structures in planar polymer substrates. J. Opt. A.

[32] Bernabeu A.P., Puerto D., Reyna J., Francés J., Márquez A., Pascual I., Gallego S., Beléndez A. (2025). Tailored adaption of thermal diffusion by ablation cooling on polymers through unique dependence on laser repetition rate. Opt. Laser Technol..

[33] Bernabeu A.P., Puerto D., Ramírez M.G., Nájar G., Francés J., Gallego S., Márquez A., Pascual I., Beléndez A. (2024). Controlled photothermal ablative processing of commercial polymers minimizing undesired thermal effects under high frequency femtosecond laser irradiation. Opt. Laser Technol..

[34] Ley C., Allona X., Di Stefano L.H., Niederst L. (2025). Pulsed laser photopolymerization: Kinetics, modeling and application in holography. Polymer.

[35] Puerto D., Gallego S., Francés J., Márquez A., Pascual I., Beléndez A. (2020). Phase-Shift Optimization in AA/PVA Photopolymers by High-Frequency Pulsed Laser. Polymers.

[36] García C., Pascual I., Costela A., García-Moreno I., Gómez C., Fimia A., Sastre R. (2002). Hologram recording in polyvinyl alcohol acrylamide photopolymers by means of pulsed laser exposure. Appl. Opt..

[37] Gallego S., Ortuño M., García C., Neipp C., Beléndez A., Pascual I. (2005). High-efficiency volume holograms recording on acrylamide and N,N′methylene-bis-acrylamide photopolymer with pulsed laser. J. Mod. Opt..

[38] Mena E.J., Bernabeu A.P., Nájar G., Gallego S., Márquez A., Beléndez A. (2025). Characterization of Holographic Gratings in PVA/AA Using Coherent Nanosecond Laser Exposure. Polymers.

[39] Gallego S., Márquez A., Méndez D., Marini S., Beléndez A., Pascual I. (2009). Spatial-phase-modulation-based study of polyvinyl-alcohol/acrylamide photopolymers in the low spatial frequency range. Appl. Opt..

[40] Liu J.M. (1982). Simple technique for measurements of pulsed Gaussian-beam spot sizes. Opt. Lett..

[41] Bernabeu A., Puerto D., Márquez A., Gallego S., Pascual I., Beléndez A. (2025). Customized PVA/AA diffraction gratings by high repetition rate femtosecond direct laser writing. SPIE Optics + Optoelectronics, Proceedings of the Holography: Advances and Modern Trends IX, Prague, Czech Republic, 7–11 April 2025.

[42] Bravo J.C., Sirvent-Verdú J.J., García-Vázquez J.C., Bernabeu A.P., Colomina-Martínez J., Fernandez R., Márquez A., Gallego S. (2024). Analysis of the recording of Fibonacci lenses on photopolymers with 3-D diffusion model. J. Eur. Opt. Soc.-Rapid Publ..

[43] Gallego S., Ortuño M., Neipp C., Garcıa C., Beléndez A., Pascual I. (2003). Overmodulation effects in volume holograms recorded on photopolymers. Opt. Commun..

[44] Gallego S., Neipp C., Ortuño M., Belendez A., Pascual I. (2004). Stabilization of volume gratings recorded in polyvinyl alcohol-acrylamide photopolymers with diffraction efficiencies higher than 90%. J. Mod. Opt..

